# Exploratory Study Comparing End-of-Life Care Intensity between Chinese American and White Advanced Cancer Patients at an American Tertiary Medical Center

**DOI:** 10.1089/pmr.2020.0064

**Published:** 2021-03-09

**Authors:** Emma Ernst, Courtney Schroeder, Avery Caz Glover, Tamara Vesel

**Affiliations:** ^1^ Tufts University School of Medicine, Boston, Massachusetts, USA.; ^2^ Division of Hematology/Oncology, Department of Medicine, Department of Medicine, Tufts Medical Center, Boston, Massachusetts, USA.; ^3^ Division of Palliative Care, Department of Medicine, Tufts Medical Center, Boston, Massachusetts, USA.

**Keywords:** advanced cancer, Chinese Americans, end-of-life care, high-intensity care, place of death, racial and ethnic disparities

## Abstract

***Background:*** Understanding ethnic disparities in end-of-life care (EOLC) intensity is central to improving outcomes for diverse populations. Although Chinese Americans represent one of the fastest growing ethnic groups in the United States, little is known about their EOLC intensity.

***Objective:*** To explore differences in indicators of high-intensity EOLC in the final 30 days of life, place of death, and hospice utilization between Chinese American and White advanced cancer patients.

***Methods:*** In this exploratory review, we collected data on 48 Chinese American and 48 White stage IV solid tumor patients who died during 2013–2018. Indicators of high-intensity care from the final 30 days of life included ≥2 hospital, ≥1 intensive care unit (ICU), and/or ≥2 emergency department admissions; cardiopulmonary resuscitation administration and mechanical ventilation (MV); place of death; and whether patients were on hospice at death.

***Results:*** Among Chinese American and White patients, respectively, 49% and 36% died in the hospital, 15% and 7% died in the ICU, 17% and 8% received MV, and 6% and 13% had ≥1 hospital admission lasting >14 days. Seventeen percent of Chinese American and 43% of White patients died at home. Hospice enrollment was similar between groups. Seventeen percent of Chinese American and 8% of White patients died within 30 days of diagnosis.

***Conclusion:*** Results suggest that fewer Chinese Americans died at home, whereas more died in the ICU, received MV, and died within 30 days of cancer diagnosis, indicating possible disparities in EOLC. Further studies are needed to explore findings from this exploratory investigation.

## Introduction

Understanding ethnic disparities in end-of-life care (EOLC) is central to improving outcomes for diverse populations. Although previous studies have explored EOLC for Asian Americans,^1^ little is known about Chinese American EOLC intensity, despite Chinese Americans representing one of the fastest growing ethnic groups in the United States.^2^

Although there are no set criteria defining EOLC intensity, researchers have identified indicators of high-intensity EOLC, including multiple hospitalizations, short intervals between hospice enrollment and death, and use of life-sustaining interventions near end of life (EOL).^3,4^ Some studies suggest that high-intensity EOLC indicators may reflect poor quality care^5,6^ and lower intensity EOLC does not decrease survival.^7^ Hospice and homecare services may decrease EOLC intensity and improve quality of life (QOL).^8–10^ Place of death may reflect EOLC quality, as death in the hospital has been associated with worse QOL and increased family psychological burden.^11^

Literature points to differences in the EOLC that Chinese Americans receive as compared with others. Asian patients are less likely to use hospice, more likely to die in the hospital,^1,12,13^ and have shorter hospice stays.^14^ Asians may be more likely to receive high-intensity EOLC and have high health care expenditures.^15^ Chinese Americans have indicated low familiarity with advance directives and hospice and preference for death in a hospital or nursing facility rather than at home.^16,17^

In this exploratory chart review, we identified specific indicators of high-intensity EOLC (adapted from past studies^1,3,6,18,19^) in the final 30 days of life, hospice enrollment, and place of death to compare Chinese American and White patients' EOLC at a tertiary medical center. We hope this exploratory study will generate hypotheses for future investigations, helping ensure that Chinese Americans receive high-quality culturally appropriate EOLC.

## Methods

We conducted an institutional review board-approved chart review of deceased Chinese American and White advanced cancer patients to compare high-intensity care indicators, hospice enrollment, and place of death. We used the hospital's cancer registry to identify all Chinese American and White stage IV solid tumor patients treated at the facility who died between January 2013 and March 2018. For each Chinese American, we matched a White patient based on timing of death per quarter year to address potential variations in documentation or care intensity over time and attain a final 1:1 ratio (48 patients per group).

We identified Chinese Americans using ethnicity codes in the cancer registry and recorded Chinese dialect spoken and use of interpretation. Data were collected on discrete high-intensity care indicators in the final 30 days including ≥2 hospital admissions, ≥1 intensive care unit (ICU) stay, ≥2 emergency department visits, and administration of cardiopulmonary resuscitation and mechanical ventilation (MV). We also collected place of death and hospice utilization. Frequencies, means, and medians were compared between groups based on clinical significance. We did not conduct statistical tests given the study's exploratory nature and lack of an a priori hypothesis.

## Results

Demographic characteristics are listed in Table 1. Thirty-three percent of Chinese American and 42% of White patients were female. Average age at death was 74 years (standard deviation [SD]: 10.8) for Chinese Americans and 69 (SD: 11.6) for White patients. Ninety-eight percent of Chinese Americans spoke a Chinese dialect and 92% required interpretation. Fifty-four percent (95% confidence interval [CI]: 39%–69%) of Chinese American and 29% (95% CI: 17%–44%) of White patients had lung cancer. Median months from diagnosis to death were 6.1 (interquartile range [IQR]: 9.3) for Chinese Americans and 9.0 (IQR: 25.0) for White patients. Seventeen percent (95% CI: 7%–30%) of Chinese American and 8% (95% CI: 2%–20%) of White patients died within 30 days of cancer diagnosis.

**Table 1.** Patient Characteristics

	Chinese American	White
Age at death, mean (SD)	74.2 ± 10.8	69.1 ± 11.6
Female, *n* (%)	16 (33)	20 (42)
Translation required, *n* (%)	44 (92)	0 (0)
Chinese dialect spoken, *n* (%)	47 (98)	0 (0)
Cantonese	36 (75)	0 (0)
Mandarin	5 (10)	0 (0)
Taishanese	4 (8)	0 (0)
Hunanese	1 (2)	0 (0)
Fuzhou	1 (2)	0 (0)
Cancer site, *n* (% [95% CI])		
Breast	0 (0 [(0–7])	4 (8 [2–20])
Gastrointestinal	17 (35 [22–51])	10 (21 [10–35])
Genitourinary	2 (4 [1–14])	8 (17 [7–30])
Gynecologic	1 (2 [0–11])	3 (6 [1–17])
Head and neck	2 (4 [1–14])	8 (17 [7–30])
Lung	26 (54 [39–69])	14 (29 [17–44])
Lung—nonsmall cell	19 (40 [26–55])	12 (25 [14–40])
Lung—small cell	6 (13 [5–25])	2 (4 [1–14])
Lung—neuroendocrine	1 (2 [0–11])	0 (0 [0–7])
Musculoskeletal	0 (0 [0–7])	1 (2 [0–11])
Months from diagnosis to death, median (IQR)	6.1 (9.3)	9.0 (25.0)
Died within 30 days of cancer diagnosis, *n* (% [95% CI]))	8 (17 [7–30])	4 (8 [2–20])

CI, confidence interval; IQR, interquartile range; SD, standard deviation.

Four of the 10 high-intensity EOLC indicators are noteworthy. Seventeen percent (95% CI: 7%–30%) of Chinese Americans received MV, 49% (95% CI: 33%–65%) died in the hospital generally and 15% (95% CI: 6%–29%) in the ICU, and 6% (95% CI: 1%–17%) had ≥1 prolonged hospital admission (>14 days) at EOL. Among White patients, 8% (95% CI: 2%–20%) received MV, 36% (95% CI: 22%–52%) died in the hospital and 7% (95% CI: 2%–19%) in the ICU, and 13% (95% CI: 5%–25%) had ≥1 prolonged hospital admission. Other indicators were similar between groups (Table 2).

**Table 2.** Indicators of High-Intensity Care

	Chinese American	White
Admissions (last 30 days), *n* (% [95% CI])		
≥2 hospital admissions	10 (21 [10–35])	10 (21 [10–35]))
≥1 hospital admission with length of stay >14 days	3 (6 [1–17])	6 (13 [5–25])
≥2 ED visits^a^	7 (16 [7–30])	5 (13 [4–27])
≥1 ICU admissions	17 (35 [22–51])	14 (29 [17–44])
Place of death^b^/hospice, *n* (% [95% CI])		
Death in hospital	20 (49 [33–65])	15 (36 [22–52])
Death in hospital ICU	6 (15 [6–29])	3 (7 [2–19])
Hospice length of stay ≤3 days^c^	6 (25 [10–27)])	8 (29 [13–49])
End-of-life intervention, *n* (% [95% CI])		
CPR (last 30 days)	1 (2 [0–11])	1 (2 [0–11])
MV (last 30 days)	8 (17 [7–30])	4 (8 [2–20])
IV chemo (last 14 days)	2 (4 [1–14])	1 (2 [0–11])

^a^ Twelve patients (4 Chinese American and 8 White) excluded from ED visit calculations due to missing data

^b^ Thirteen patients (7 Chinese American and 6 White) excluded from place of death calculations due to missing data

^c^ Percentages calculated out of 52 patients enrolled in hospice. One hospice length of stay was unknown and excluded from the calculation.

CPR, cardiopulmonary resuscitation; ED, emergency department; ICU, intensive care unit; IV, intravenous; MV, mechanical ventilation.

Hospice data and place of death are listed in Table 3. Fifty percent (95% CI: 35%–65%) of Chinese American and 60% (95% CI: 45%–74%) of White patients were enrolled in hospice. Median hospice enrollment was 18 days (IQR: 39.8) for Chinese American and 9 days (IQR: 19.0) for White patients. Seventeen percent (95% CI: 7%–32%) of Chinese American and 43% (95% CI: 28%–59%) of White patients died at home.

**Table 3.** Hospice and Place of Death

	Chinese American	White
Hospice enrollment, *n* (% [95% CI])	24 (50 [35–65])	29 (60 [45–74])
Hospice length of stay for patients enrolled in hospice, median (IQR)	18 (39.8)	9 (19)
Place of death, *n* (% [95% CI])		
Home	7 (17 [7–32])	18 (43 [28–59])
Hospital	20 (49 [33–65])	15 (36 [22–52])
Nursing home	7 (17 [7–32])	2 (5 [1–16])
Inpatient hospice facility	7 (17 [7–32])	7 (17 [7–31])
Total	41 (100)	42 (100)
Place unknown	7	6

Unknowns excluded from percentages and calculations.

## Discussion

To our knowledge, this exploratory study is the first comparing EOLC intensity among Chinese American and White advanced cancer patients. Our findings highlight potential disparities or differences in preference that deserve further inquiry.

Our findings suggest that Chinese Americans died earlier after diagnosis. This may be related to older age and a higher proportion of lung cancer among the Chinese Americans. Another consideration could be disparities in the care that Chinese Americans receive due to cultural, socioeconomic, or language barriers.

The results suggest that measures of high-intensity EOLC were similar between the groups with the exception of higher rates of MV and death in the hospital and ICU among Chinese Americans and a higher rate of prolonged hospital admissions among White patients. Higher MV rates among Chinese patients, as suggested here and recently shown elsewhere,^20^ may be attributed to low familiarity with advance directives^17,21^ or reluctance to discuss EOL due to concerns that such conversations bring bad luck.^22^ It may also be linked to the expectation of filial piety among Chinese families, which emphasizes children's duty to care for parents and may lead children to advocate for life-sustaining treatments for their parents.^21,23^ Still, recent findings suggest that high MV rates may not reflect Chinese individuals' wishes.^24^ Improved advance care planning, which Chinese patients may be more receptive to than previously thought,^25^ could clarify discrepancies.

We found the most notable difference between groups in place of death. Similar findings have been noted previously^1,13,16^ and research has suggested that Chinese Americans do not prefer to die at home.^22,26^ When Chinese Americans were asked to rank priorities for EOL, none ranked “to die at home.”^26^ Older Chinese adults have expressed a preference for death in the hospital, suggesting that it helps a dying person maintain hope and makes it easier for family to visit and offer support, while home deaths might negatively impact house resale value or cause “contamination.”^27^ Although research on traditional death in Chinese culture notes that “dying at home is a way of continuing bonds with ancestors,”^22^ this practice has become less common in China. Reasons include a lack of resources to support EOL home care, congested living environments, and home death being viewed as bad luck.^22^ More single-generation households in China mean decreased family support for home death^28^ and patients may be reluctant to die at home if they perceive it as a burden on family.^16^ The preference for death outside the home has important clinical implications for EOL planning. Although evidence suggests that most people favor death at home,^29^ health care providers should know that Chinese Americans may not share this preference.

Our findings suggesting that half of Chinese Americans in the sample were enrolled in hospice and had potentially longer hospice stays differ from past findings that Asian Americans, and Chinese Americans in particular, are less likely to accept hospice.^1,13,16^ Our results may reflect an evolution in Chinese American views, though further research is needed.

Strengths of this investigation include the fact that it focuses on an understudied topic and highlights potential disparities in EOLC that deserve further investigation. Limitations include a small sample size from a single medical center, restricting generalizability. We were unable to capture additional demographic characteristics such as religious affiliation, country of birth, and years in the United States,^30^ though future studies should consider such factors. Findings may be confounded by demographic and clinical differences, including older age and a higher proportion of lung cancer among the Chinese American patients. Although study characteristics and EOLC intensity indicators used are consistent with prior research (retrospective, cancer patients, 30-day EOL timeframe), these measures and EOL timeframe currently lack validation and researcher consensus.^3^ Since this was an exploratory study, further investigations are needed to confirm and understand findings.

## Conclusion

Our exploratory results suggested that higher rates of Chinese American advanced cancer patients died within 30 days of diagnosis, received MV near EOL, and died in the hospital and ICU though they had lower rates of prolonged hospital admissions than White patients. Other high-intensity EOLC indicators were similar between groups. Results also suggest low rates of home death among Chinese Americans, despite a higher hospice enrollment rate than noted for Asian Americans previously. Robust studies are needed to further characterize differences and investigate the source of discrepancies in EOLC intensity and place of death, so that higher quality culturally appropriate EOLC may be provided to Chinese American advanced cancer patients in the future.

## Abbreviations Used

CI: confidence interval
CPR: cardiopulmonary resuscitation
ED: emergency department
EOL: end of life
EOLC: end-of-life care
ICU: intensive care unit
IQR: interquartile range
MV: mechanical ventilation
QOL: quality of life
SD: standard deviation
